# Mobility Disability and Exercise: Health Outcomes of an Accessible Community-Based Center

**DOI:** 10.3389/fresc.2022.836655

**Published:** 2022-03-23

**Authors:** Kerri A. Morgan, Kelly L. Taylor, Carla Wilson Walker, Susan Tucker, Jessica L. Dashner, Holly Hollingsworth

**Affiliations:** ^1^Enabling Mobility in the Community Laboratory, Program in Occupational Therapy, Washington University School of Medicine, St. Louis, MO, United States; ^2^Occupational Therapy Program, Murray State University, Paducah, KY, United States

**Keywords:** exercise, mobility disability, strength, endurance, community-based research

## Abstract

**Objective:**

The purpose of this study was to determine how support and guidance provided by trained professionals during a 12-week, community-based transition exercise program, impact health outcomes and continued engagement in physical activity for persons with a mobility disability (PwMD).

**Design:**

A single arm pre-post design was used.

**Setting:**

Accessible community-based health and wellness center.

**Participants:**

The study included 244 PwMD using a mobility device.

**Interventions:**

Participants completed a 12-week transition exercise program provided through an accessible community facility that provided education and support to complete endurance and strength related exercises as well as programming to encourage transition to self-directed engagement in exercise.

**Main Outcome Measures:**

Bodyweight, BMI, pain, perceived exertion, speed, and distance during cardiovascular fitness testing, and strength were measured pre and post exercise program. The number of participants that signed up for a monthly membership after the program was also monitored.

**Results:**

For the total group, average pain reported over previous 30 days decreased significantly (*p* < 0.01), current daily pain decreased significantly (*p* < 0.05), perceived exertion at the end of the 9-min endurance test decreased significantly (*p* < 0.05), and the four upper extremity strength exercises showed large, significant strength gains (*p* < 0.01) after the program. There was no significant change in bodyweight, BMI, or speed and distance completed during endurance testing. At the completion of the program, 76% of participants enrolled in a monthly membership at the facility with the intentions to continue to exercise regularly.

**Conclusions:**

This study provides evidence that an accessible community-based exercise program, with a transitional component supported by trained professionals, can support the exercise goals of PwMD and improve strength, decrease pain, and may promote regular exercise adoption for PwMD.

## Introduction

According to data from the National Health Interview Survey, 5.8% of Americans aged 18–64 years have a mobility disability (1). Persons with a mobility disability (PwMD) are at a greater risk for major health conditions including cardiovascular disease, hypertension, and diabetes compared to those without disabilities (2–4). One of the major contributors to these health disparities experienced by PwMD is that they are more vulnerable to secondary conditions that result in an increased number of hospitalizations and high costs for treatments that could be prevented through improved levels of exercise. The number of PwMD will continue to grow, as the number of people using wheelchairs is predicted to quadruple between 2005 and 2030 (5). Compared to other disability groups (vision, hearing, and cognition), adults with a mobility disability have the highest prevalence of inactivity, with over half reporting inactivity (1).

Regular exercise is widely recognized as having health benefits (2, 6–8) and is also connected with more established social networks, greater participation in life activities, and greater likelihood of employment (9–11). However, PwMD remain one of the least physically active populations in the U.S., and those who are active, often are not experiencing the health-related benefits of exercise (12, 13). The typical daily routine of PwMD does not produce positive health-related changes such as cardiovascular increases; therefore, structured exercise activities are needed to promote health-related benefits (14). Consistent participation in exercise is a difficult area of reintegration for PwMD outside of the traditional clinical setting (15), with decline in functional capacity often occurring after discharge from rehabilitation (9). A gap in the continuum of care exists from rehabilitation to the community; therefore, PwMD often lack the appropriate guidance and resources to achieve successful exercise goals following completion of therapy (10).

PwMD commonly experience physical or program barriers that limit or prevent them from accessing health and wellness programs outside of the medical and rehabilitation model (10, 16). The barriers to participating in regular, structured exercise, outside the medical and rehabilitation setting, are well documented for PwMD. Environmental barriers to exercise for PwMD include considerable lack of accessible facilities, equipment, supports, and lack of trained, knowledgeable staff (10, 15, 17–22). Personal barriers to exercise participation include lack of information on available exercise programs and accessible facilities in the community; lack of experience with exercise equipment, programming and techniques; and reduced motivation to voluntarily participate in physical exertion. PwMD have also reported being inundated regarding initiating an exercise program, especially if they were not familiar with exercise techniques prior to their disability (17, 18, 23, 24). PwMD may also experience a perceived conflict with the cultural norms of traditional community gyms, leading to negative interactions with staff and other gym participants (23).

Traditional home exercise programs, often prescribed at discharge from rehabilitation, may overcome initial participation barriers but often do not provide support for initiating an exercise program designed to promote health-related benefits, supervision during physical activity, ongoing education or an optimally individualized physical activity prescription, which improves outcomes, progression, and safety monitoring (25). Researchers continue to explore methods and strategies to engage PwMD in physical activity, and common shortfalls of these approaches include a lack of professional support, poor adherence rates, inability to maintain physical activity increases, and tested interventions not translating into sustainable models (25, 26). Thus, transition from clinical evidence of exercise effectiveness to provision and establishment of effective community-based exercise programs (CBEPs) for PwMD has proven challenging. More evidence is needed to identify evidence-based exercise approaches implemented in the community that improve the participation of exercise for underserved PwMD (15).

To address barriers and promote physical activity participation among community-dwelling PwMD, CBEPs need an adequate combination of participant education; individualized programming based on evidence-based recommendations; knowledgeable, trained support staff (11); and accessible equipment within an accessible facility. CBEPs may be an essential component in the continuum of care to monitor and optimize health, function, and participation for PwMD (10). Furthermore, it is worthwhile to determine how to best support PwMD to establish routines as life-long exercisers, prevent secondary health conditions, and promote their overall well-being. Therefore, the purposes of our study were to determine (1) the prevalence of participants who remained engaged in regular exercise following completion of a 12-week, community-based, transition exercise program and (2) health-related outcomes of participating in the 12-week CBEP for PwMD. Our study seeks to fill a gap in the literature regarding health outcomes and exercise engagement for PwMD successfully participating in supportive, accessible CBEP.

## Materials and Methods

### Study Design

A pre-post, single arm, prospective, within-subject design was used.

### Setting

The long-running research study took place from March 2006 to October 2017 at an accessible exercise facility in the Midwest region of the United States operated by a disability organization. The accessible exercise facility component of the organization was staffed by trained healthcare professionals including occupational therapists, occupational therapy assistants, and a physical therapy assistant. The organization also has relationships with several local higher education institutions, from which the exercise facility accepts health professional graduate students for clinical fieldwork rotations, regular community service engagement, and other capacities.

### Participants

Potential participants were recruited *via* flyers, advertisements, referrals from local rehabilitation facilities, and word-of-mouth. Participants were eligible for inclusion if they: (1) were 18 years or older, (2) had a mobility disability requiring use of a mobility device, (3) were community-dwelling, and (4) were able to provide informed consent. The International Classification of Functioning, Disability and Health (ICF) model was used to frame the basis for participant eligibility related to mobility disability instead of medical diagnosis (27). The following ICF codes were used as inclusion criteria to recruit and enroll a non-representative convenience sample of PwMD: b730 Muscle power function, d450 Walking exclusion, d465 Moving around using equipment, and e120 Use assistive mobility device (28). The project was approved by our university institutional review board. Persons were excluded from the study if they did not have a medical condition requiring use of a mobility device (cane/crutch(s)/walker, manual wheelchair, power wheelchair, scooter); were under 18 years of age, lived in a facility such as a nursing home, were unable to provide informed consent, or unable to provide a physician release to exercise.

### Community-Based Exercise Intervention Program

The 12-week exercise program consisted of 1:1 or 2:1 guided and supervised exercise training by trained staff. Staff included occupational therapists, an occupational therapy assistant, a physical therapy assistant, and graduate students from healthcare programs. The exercise program was based on the American College of Sports Medicine's Physical Activity Recommendations (29, 30). The intervention included 1–2 h sessions, one-to-three times per week, for a goal of 12 weeks. Prior to each exercise session, participants' current pain level was recorded. While the 12-week program was centered on each participant's goals, abilities, and preferences, exercise sessions maintained a foundational structure. Each exercise session included a warm-up, opportunities to do cardiovascular and strength exercises and a cool-down. Many participants also chose to do flexibility training during their sessions through range-of-motion and stretching exercises.

The primary goal of the program was for participants to self-direct their own exercise regimens following the 12-week program. The exercise program followed three adaptable phases that progressed at varying rates depending on individual participants: (1) education and setup, (2) guidance and assistance, and (3) transition and monitoring. During the education and setup phase, staff educated participants on physical activity recommendations and various exercise modes, and provided instruction and demonstration on proper equipment setup and exercise technique. The majority of the exercise program consisted of the guidance and assistance phase. During this phase, staff provided verbal, visual, and/or physical support to assist participants during their exercise sessions including transfers, equipment setup, spotting, or adjusting exercise techniques. The transition and monitoring phase occurred throughout the program but became the focus during the final 2–3 weeks. Staff provided less guidance during exercise sessions, promoting participants' autonomy and self-monitoring. For example, participants might prefer more cardiovascular workouts to achieve their goals and choose to complete both the Vitaglide and the arm ergometer while only completing a few of the strengthening exercises (biceps, rickshaw).

To ensure fidelity and consistency, study protocol and procedures were maintained throughout the study. During the time period of this study, changes included addition of new equipment and a few changes in staffing. Quarterly staff trainings were conducted to ensure that all staff were consistent with testing and intervention protocol and procedures. To maintain data collection fidelity, testing and workout tracking forms were employed to guide staff on protocol delivery and data documentation.

### Exercise Equipment

Participants used a variety of equipment during the 12-week exercise program. Strength equipment included the Uppertone (GPK Inc., El Cajon, CA, USA), Equalizer (Equalizer Exercise Machines, Red Deer, Alberta, CA), free weights, and resistance bands. Endurance equipment included the Endorphin Arm Ergometer (ArmE; Pro-Med Products, Alpharetta, GA, USA), Motomed (RECK-Technik GmbH & Co., Betzenweiler, DE), Vitaglide (Planet Mobility, Shelby Township, MI, USA), manual wheelchair rollers (provided by Dr. Rory A. Cooper, PhD, University of Pittsburgh, Pittsburgh, PA, USA), standard treadmill (Planet Mobility, Shelby Township, MI), and NuStep (NuStep, LLC, Ann Arbor, MI, USA). All exercise equipment used for assessments and exercise sessions was accessible for PwMD. During assessments, participants were tested to measure their baseline strength using either the Uppertone or Equalizer, and tested to measure their baseline endurance using the ArmE. During exercise sessions, participants had access to any of the available equipment in the facility. Descriptions of the equipment can be found in Appendix A.

### Procedures

Eligible participants attended a workshop, where they were screened for eligibility, given information on the facility and the 12-week exercise program, toured the facility, and provided informed consent. Enrolled participants were required to obtain physician's release prior to beginning exercise.

#### Testing Protocol

All study participants completed baseline (T-1) testing prior to and terminal (T-2) testing at the completion of the 12-week program. Bodyweight, resting vitals, and current and average pain were measured using assessment equipment and established outcome measures below.

The cardiovascular endurance test was performed with the upper extremities on the ArmE. Participants transferred to a seat or sat in their mobility devices to perform the test. For participants who were unable to grip the ArmE handles, grip assists, Ace bandage wraps, or neoprene gloves were used to secure their hands. The asynchronous ArmE protocol included an initial 30-s speed test to establish a testing speed the participant perceived as “hard,” or 5/10 on the Modified Borg RPE scale (31). The speed test was followed by the 9-min graded exercise test, in which participants were instructed to maintain the established “hard” speed throughout the test. All participants initiated the test at 19 W, with incremental increases by 4 W every 3 min for 9 min. Participants were asked to rate their perceived exertion (1–10) (32) at the end of minutes three, six, and nine and following completion of the test.

Strength testing consisted of establishing a 1-repeition-maximum (1-RM) on four upper extremity exercises (chest press, back row, biceps curl, and rickshaw triceps extension) performed unilaterally. The highest amount of weight pushed or pulled through a complete range of motion was recorded as the 1-RM using the Uppertone, or Equalizer.

### Outcome Measures

Cardiorespiratory fitness was assessed using a 9-min, incremental test with the ArmE by measuring average speed at minutes three and nine (m/s), self-reported perceived exertion of the 3rd and 9th min (RPE; 1–10) (32) and total distance completed (m). Strength was assessed *via* unilateral 1-RM (kg) across four upper extremity exercises (chest press, back row, biceps curl, and rickshaw) unilaterally with the right upper extremity, using either the Uppertone or Equalizer. Weight was measured using a Seca model 664 digital wheelchair scale. The average level of pain for the previous 30 days was assessed at each assessment using the Wong-Baker FACES Pain Rating Scale (0–10) (33, 34). The Characteristics of Respondents survey (CORE) (35) was also used to gather demographic information (Table 1). Interest in continuing to exercise was measured by whether or not the participant signed up for a monthly membership to the facility following completion of the program.

**Table 1:** Assessments made prior to (T-1) and after T-2) exercise intervention.

**Measure**	**Instrument/device**	**Unit of measure**
Demographics	CORE survey	
Fitness
Body Weight	Seca model 664 wheelchair scale	pounds (converted to kg)
Body mass index (BMI)	Calculated	weight/height
Current pain level	Faces Pain Rating Scale	1–10 (high)
Average pain level over past 30 days	CORE	1–4 (high)
Endurance
Speed of arm crank turn (ArmE)–3 min	Endorphin^®^ Arm/Leg Ergometer	meters/second
Speed of arm crank turn (ArmE)–9 min	Endorphin^®^ Arm/Leg Ergometer	meters/second
Rate of perceived exertion (RPE)–3 min	Modified Borg Scale	1 to 10 (high)
Rate of perceived exertion (RPE)–9 min	Modified Borg Scale	1 to 10 (high)
Strength/resistance
Biceps	Uppertone or Equalizer	repetition maximum (1-RM)
Chest press	Uppertone or Equalizer	repetition maximum (1-RM)
Rickshaw triceps extension	Uppertone or Equalizer	repetition maximum (1-RM)
Rowing left	Uppertone or Equalizer	repetition maximum (1-RM)

*ArmE, arm ergometer*.

### Data Analysis

Statistical analyses were conducted using IBM SPSS Statistics (Version 26). Demographic information was compiled using descriptive statistical methods. Paired sample *t*-tests were used to compare outcome measures (bodyweight, current pain, average pain over 30 days, cardiovascular fitness, and strength) between baseline and terminal assessments. Dependent measures for cardiorespiratory fitness included average speed and RPE during the 3rd and 9th min and total distance completed. Equipment used to perform the strength testing protocol changed from the Uppertone to the Equalizer in October 2009. Therefore, strength data were separated and compared according to equipment used. Pre-post 1-RM for each of the four upper extremity exercises using the right arm, were used as dependent measures to determine changes in strength. Statistical comparisons were conducted for the total group, as well as between session frequency intensities (intermittent v. concentrated). *Intermittent* participants completed an average of one session per week, while *concentrated* participants completed an average of two to three sessions per week. Outcome measures were also compared among the three most frequent diagnoses: spinal cord injury, stroke, and multiple sclerosis. Due to mitigating factors impacting many participants' ability to complete 2–3 sessions per week, program duration inclusion was expanded to include participants who completed the CBEP in 12–18 weeks with a minimum of ten sessions and maximum of 36 sessions. Values are expressed as mean ± SD, unless otherwise stated. Two-tailed significance was accepted at *p* < 0.05.

## Results

**Figure 1:**
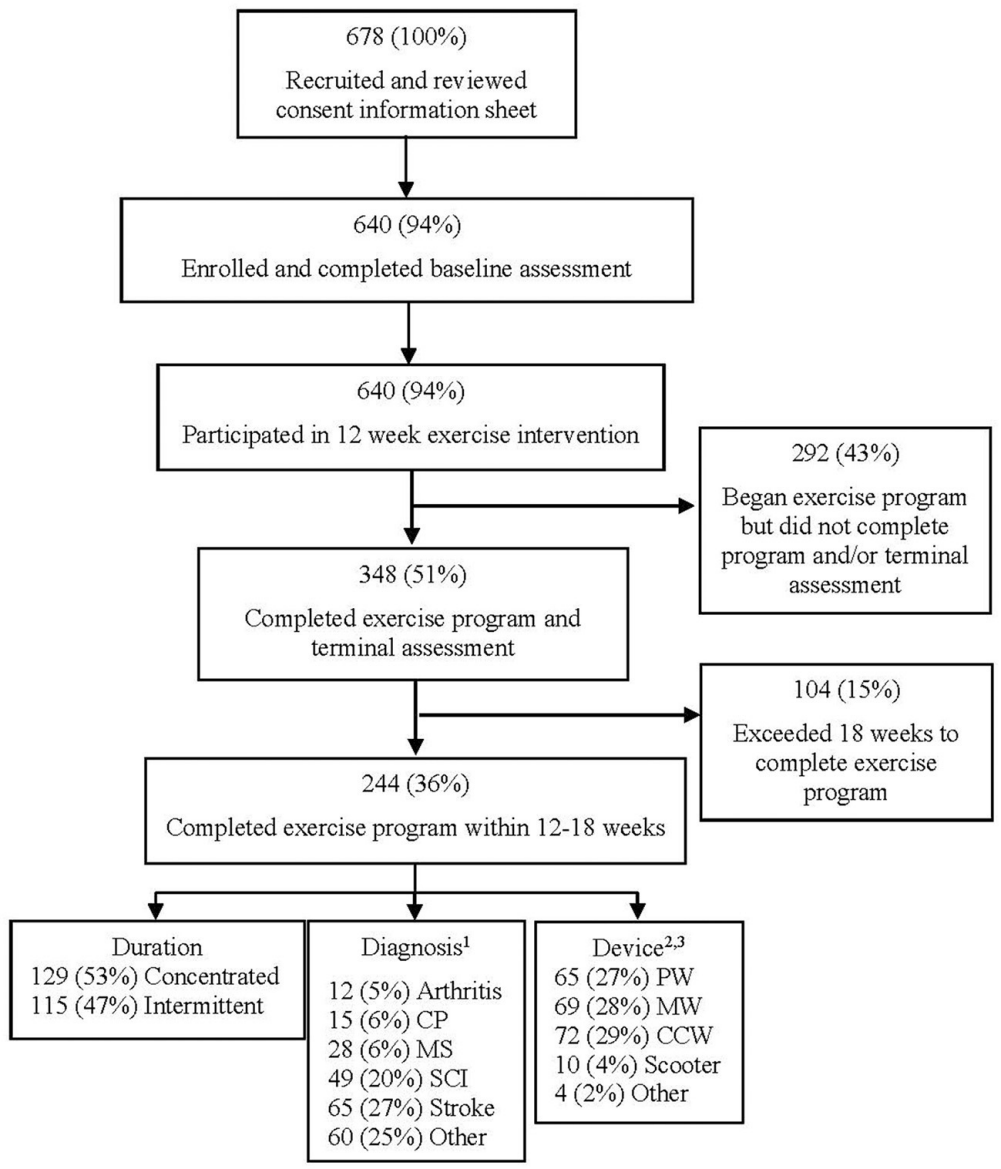
Participant recruitment, enrollment, and study participation flow diagram. ^1^Refused to answer the diagnosis survey question (*n* = 15, 11%). ^2^PW—power wheelchair, MW—manual wheelchair, CCW—cane, crutches, and walkers. ^3^Refused to answer the device-used survey question (*n* = 24, 10%).

Over the course of the long-running CBEP study, 348 participants completed the 12-week program and both testing sessions and 244 of these participants met the eligibility criteria for analysis (Figure 1). Two hundred forty-four participants completed both assessments and finished the 12-week exercise program within 12–18 weeks (Table 2). There was equal representation of gender and nearly equal of race (48.8% White and 41.8% non-white) with the majority being low income and fairly high levels of education. A mean 20.4 ± 5.5 (range 10–34) total sessions were completed over an average 13.5 ± 1.8 weeks. Weekly frequency of exercise sessions varied from one to three, with an average 1.54 ± 0.5 sessions per week. Out of 244 participants, 76% (*n* = 186) expressed interest in continuing to exercise by enrolling in the monthly membership program at the facility.

**Table 2:** Demography of intervention sample (*N* = 244).

	**Mean (SD)**	**Range**
Age (241 responded)	51.12 (15.41)	17-88
Gender	***n***	**%**
Female	121	49.6
Male	121	49.6
No answer	2	0.8
Race
Black/African American	102	41.8
White	119	48.8
Other	14	5.7
No answer	3	1.2
Personal annual income
$0–$14,999	101	41.4
$15,000–$34,999	62	25.4
$35,000–$54,999	17	7.0
$55,000 or more	16	6.5
No answer	48	19.7
Highest grade completed
Grade 1–11	23	9.4
Grade 12/GED	59	24.2
College 1–3 years	75	30.7
College ≥ 4 years	80	32.8
No answer	7	2.8
Primary disability
Arthritis	12	4.9
Cerebral palsy	15	6.1
Multiple sclerosis—MS	28	11.5
Spinal cord injury—SCI	49	20.1
Stroke	65	26.6
Other conditions	60	24.5
No answer	15	6.1
Device
Power wheelchair	65	26.6
Manual wheelchair	69	28.3
Cane/crutches/walker	72	29.4
Scooter	10	4.1
Other devices	4	1.6
No answer	24	9.8

*SD, standard deviation; GED, general education diploma*.

Total group results (*n* = 244; Table 3) of the 12-week exercise program showed no significant changes in bodyweight or BMI. Current pain and 30-day average pain decreased significantly (*p* < 0.05 and *p* < 0.01, respectively). A statistically significant decrease in RPE was achieved at minute-nine of the ArmE endurance test (*p* < 0.05). Total distance completed on ArmE endurance test increased by 11%; however, this was not significant. Strength significantly increased across all four strength exercises regardless of equipment used. Increases in strength for all exercises exceeded 10%. No significant differences were found between workout frequency intensities or among diagnoses.

**Table 3:** Influence of exercise on total intervention group (*N* = 244).

**Variables**	***n***	**T-1 Mean (SD)**	**T-2 Mean (SD)**	**T-2-T-1**	**%Δ**
Body weight (kg)	134	85.0 (28.6)	85.8 (27.5)	0.8	0.9
BMI	130	29.5 (9.3)	29.8 (9.0)	0.3	1.0
Current pain	230	2.1 (2.6)	1.7 (2.3)	−0.4	−23.5^*^
Average pain	223	3.2 (2.7)	2.3 (2.9)	−0.9	−39.1^†^
Arm ergometer (ArmE)
ArmE speed: 3 min (m/s)	214	7.0 (4.1)	7.2 (3.2)	0.2	2.7
ArmE speed: 9 min (m/s)	203	5.7 (3.9)	5.7 (3.0)	0.0	0.0
ArmE RPE: 3 min	232	3.2 (1.7)	3.2 (1.7)	0.0	0.0
ArmE RPE: 9 min	219	5.6 (2.1)	5.3 (2.1)	−0.3	−5.7^*^
ArmE total distance (km)	211	1.6 (1.0)	1.8 (3.2)	0.2	11.1
Uppertone (*n =* 96)
Biceps (kg)	81	13.7 (9.1)	15.3 (9.3)	1.6	10.4^†^
Chest (kg)	88	22.8 (11.2)	26.8 (10.4)	4.0	14.9^†^
Rickshaw (kg)	86	16.6 (8.2)	20.9 (9.5)	4.3	20.6^†^
Rowing (kg)	90	23.0 (10.2)	28.0 (9.6)	5.0	17.9^†^
Equalizer (*n =* 148)
Biceps (kg)	62	10.7 (6.8)	13.0 (7.1)	2.3	17.7^†^
Chest (kg)	76	15.5 (10.2)	21.0 (11.9)	5.5	26.2^†^
Rickshaw (kg)	128	23.0 (13.4)	30.5 (16.7)	7.5	24.6^†^
Rowing (kg)	76	20.0 (9.7)	26.0 (12.3)	6.0	23.1^†^

*SD, standard deviation; ArmE, arm ergometer; min, minutes; m/s, meters per second; kg, kilograms; km, kilometers,*

* *p < 0.05,*

† *p < 0.01*.

## Discussion

Although exercise intervention research for people with disabilities has become more prevalent in the last decade [increasing by 60% since 2010; (36)], most studies are diagnosis-specific, significantly limiting sample size and reducing generalizability to the overarching population of PwMD. Most CBEPs are also limited by often targeting ambulatory populations, lack of customizable programming for neurological diagnoses other than stroke, and little structure for individuals to continue exercising after the formal intervention period is complete (9). The current study sought to provide a staff-guided, multi-modal transitional exercise program in an accessible, community-based facility and assess effectiveness in improving health-related outcomes and to promote adoption of a physically active lifestyle among PwMD who often cannot successfully utilize traditional fitness centers and gyms.

Providing direct professional support and guidance alone may not directly result in long-term behavior change. Interventions rooted in self-determination theory (SDT), which focus on cultivating autonomous motivation, a sense of belongingness, and confidence in one's actions, have shown promise in promoting physical activity—related behavior change (37, 38). Similarly, behavior change techniques (BCT), or systematic intervention methods used to change psychological determinants of behavior (e.g., self-efficacy, health beliefs), are commonly used in interventions for physical activity for spinal cord injury research. The theoretical mechanism of action for self-management is self-efficacy, which was promoted in this study *via* the three-phase transitional CBEP, particularly the transition and monitoring phase, may have contributed to a high percentage of participants wanting to continue to exercise (39).

Evaluation of behavior change is becoming more prevalent related to healthy lifestyle adoption among PwMD (36). Interventions that support autonomy and self-efficacy in exercise participation have been shown to promote self-management and increase the probability of implementing physically active behaviors, independently (40). The current study integrated a transition and monitoring phase to support participants' autonomy and ownership over their exercise routines to promote continuation of regular physical activity participation. Seventy-six percent of participants successfully transitioned and maintained their exercise regimens following completion of the 12-week program. The transitional component of an exercise program is critically important to lifelong engagement in physical activity for PwMD (9). The adoption of an ongoing physical activity regime has been shown to decrease secondary conditions and improve overall health, thus making the investigation of the successes found in this CBEP important to analyze in order to determine how to replicate widely.

Large, significant increases in strength occurred across all four exercises, which align with previous research (41–43) and further support the musculoskeletal benefits of consistent participation in structured, individualized strength training in CBEPs for PwMD. Participants demonstrated a mean increase of over 5.9 kg (23%) in strength for back row and rickshaw triceps extension exercises. Improved performance on these two exercises is particularly important for individuals using wheeled mobility devices, as they counterbalance muscles frequently used for wheelchair propulsion, as well as strengthen muscles used during functional transfers (44).

Minimal changes in cardiorespiratory fitness occurred during the study, with a decrease in RPE for minutes-three of the ArmE endurance test being the only significant result. These results differ from previous clinical studies of endurance exercise interventions, which have shown improved cardiorespiratory fitness (36, 42, 43, 45). Following recommended guidelines (30), measurable increases in cardiovascular health can occur within 8–12 weeks (46–49); however, a minimum exercise intensity threshold must be met. Exercise intensity is a key component for changes in aerobic capacity, with moderate-to-vigorous intensity being the recommended threshold for the frequency and duration of the current study (30). Intensity was only measured during assessments and was not regulated during exercise sessions.

Minimal change in cardiorespiratory fitness may also be explained by methodological limitations. Peak oxygen consumption (VO_2peak_) during a graded exercise test is currently the gold-standard for assessing cardiorespiratory fitness and is often used in studies examining endurance changes in PwMD (50, 51). VO_2peak_ testing assesses changes at the metabolic level, providing greater sensitivity to changes compared to the ArmE endurance testing protocol administered in this study. Previous studies that used similarly broad endurance testing methods reported minimal-to-no significant changes in cardiorespiratory endurance (9, 11). CBEPs typically do not have access to the equipment and advanced training required to reliably conduct metabolic testing. Despite the lack of change in cardiorespiratory fitness, the significant decrease in RPE indicates reduction of perceived effort at the same exercise intensity, which may be an indicator of improved endurance. The CBEP, described in the current study, promoted self-efficacy among participants with the objective of improving self-directed participation in an exercise program. Previous evidence supports the relationship between self-efficacy and improved changes in RPE (52).

Varied dosing-frequency may have also contributed to limited changes in cardiorespiratory fitness. While the curvature of the dose-response relationship remains ambiguous, numerous studies have concluded the health benefits of regular physical activity (53) directed by pre-established guidelines (4, 25). Similar to exercise intensity, a minimum frequency threshold must be met to elicit measurable improvements in cardiorespiratory fitness. Intermittent participants (average of one session/week) comprised 47% of our included sample; as the recommended frequency of moderate-vigorous cardiovascular exercise is 3–5 days per week (4, 25), these intermittent participants likely did not meet the minimum threshold to produce measurable improvements in cardiorespiratory health.

No significant changes in weight were noted, similar to some previous studies (11, 54–56) and different from others (47). Previous studies found similar results related to BMI (54, 57). Pain (both current and average over previous 30 days) decreased significantly. Limited evidence exists examining the effects of exercise on pain reduction for PwMD; however, available literature supports the present study's findings in acute and chronic pain reductions post-exercise intervention (58–61).

### Study Limitations and Future Directions

The current study included limitations in methodological rigor and outcome measures. This study lacked a control group limiting the causative inferences of the CBEP. The lack of changes in cardiorespiratory fitness may reflect the inadequate sensitivity of the measures used. While this is a limiting factor of the study, practicality should be considered for community-based settings; future disability and physical activity research should explore alternative methods for measuring fitness that are reliable and sensitive but also practical in a community-based setting, as this aligns with current National Institute of Health recommendations (62).

One of the primary objectives of this study was to determine the prevalence of participants who remained engaged in regular exercise after completing the CBEP, which yielded a 76% success rate. The current study did not include a formal outcome measure of self-efficacy to attempt at measuring continued success outside of the CBEP such as quantitative questionnaires or qualitative interviews. Future studies should utilize such assessment tools to evaluate self-efficacy and autonomy at baseline and at distinct timepoints throughout participation in exercise to future guide development of CBEPs for PwMD.

Self-direction is a fundamental difference between clinic-based exercise programs and CBEPs. CBEPs, like the present study, allow participants to select exercise equipment based on their personal goals and preferences, resulting in increased variability of equipment used and exercises performed. For example, endurance exercises had no specific time, distance, or intensity per session and were variable by participant. In contrast, for strength exercises, support staff often set a distinct number of sets and repetitions at specified weights for participants. A dose-response analysis was conducted based on weekly frequency, but no significant differences were found, likely confounded by the variability of exercises completed across participants. Weekly frequency was also often dependent on several variables including transportation availability, health status, personal assistance and work schedules, and support staff availability. Time to complete the 12-week program also varied to accommodate for mitigating factors impacting many participants' ability to complete two to three exercise sessions every week. Due to the importance of individualization of exercise programming for PwMD, future studies should incorporate reliable and sensitive methods for tracking intensity during testing and intervention to further customize participants' exercise frequency and duration based on intensity achieved. Future studies should also consider tracking participants' daily activity outside of the exercise sessions to examine any changes or differences in activity patterns in PwMD's daily lives.

## Conclusions

Exercise is one of the vehicles for community reintegration and participation for PwMD; however, physical inactivity remains one of the hallmark traits of this population (63). This study provides evidence that an individualized CBEP can significantly improve upper extremity strength and decrease pain for PwMD, as well as effectively transition PwMD from a formally guided program to a self-monitoring continuation of regular physical activity. The program in this study provided knowledgeable, professional support; customized programming; and an accessible facility and equipment, which are integral for PwMD to participate in CBEPs. Lack of change in cardiorespiratory fitness was likely attributed to methodological limitations of the endurance test, inadequate achievement of minimally-recommended exercise intensity and frequency, and decreased sensitivity of the methods used to assess fitness and monitor intervention intensity. PwMD require access to accessible, community-based fitness programs post-rehabilitation to continue recovery, reduce risk of comorbidities and mortality, and optimize functional independence, societal participation, and overall quality of life.

## Data Availability Statement

The original contributions presented in the study are included in the article/Supplementary Material, further inquiries can be directed to the corresponding author/s.

## Ethics Statement

The studies involving human participants were reviewed and approved by Washington University Institutional Review Board. The patients/participants provided their written informed consent to participate in this study.

## Author Contributions

KM assisted with original conception and design of the study and assisted with data analysis and interpretation of the data. She drafted the article and provided final approval to the draft being submitted for publication. KT was involved with analyzing and interpreting the data. She provided intellectual content related to all sections of the paper and provided final approval to the draft being submitted for publication. CW was involved with providing technological support to the acquisition of data. She contributed intellectual content to the methods, results and discussion sections and provided final approval to the draft being submitted for publication. ST and JD were involved with data collection. They contributed intellectual content to the methods section and provided final approval to the draft being submitted for publication. HH was involved with designing the study, data analysis and interpretation of the data. He contributed intellectual content to the methods and results sections and provided final approval to the draft being submitted for publication. All authors contributed to the article and approved the submitted version.

## Funding

Missouri Foundation for Health (04-0551); US Department of Education, NIDRR (H133A010701); and National Institutes of Health, Comprehensive Opportunities in Rehabilitation Research Training (K12 HD055931).

## Conflict of Interest

The authors declare that the research was conducted in the absence of any commercial or financial relationships that could be construed as a potential conflict of interest.

## Publisher's Note

All claims expressed in this article are solely those of the authors and do not necessarily represent those of their affiliated organizations, or those of the publisher, the editors and the reviewers. Any product that may be evaluated in this article, or claim that may be made by its manufacturer, is not guaranteed or endorsed by the publisher.

## Abbreviations

1-RM: 1-repetition maximum
ArmE: Arm/Leg Ergometer
CBEP: Community-based exercise program
CORE: Characteristics of Respondents survey
ICF, International Classification of Functioning: Disability and Health
PwMD: Persons with a mobility disability
RPE: Rating of perceived exertion
SCI: Spinal cord injury
T-1: Baseline assessment
T-2: Terminal assessment
VO_2peak_Peak oxygen consumption
W: watts.
