# Supplementary material for: Mobility Disability and Exercise: Health Outcomes of an Accessible Community-Based Center
Source: Front Rehabil Sci. 2022 Mar 23;3:836655. doi: 10.3389/fresc.2022.836655 (PMC9397740; doi:10.3389/fresc.2022.836655)
Supplement: Supplementary file 1 [file Table_1.DOCX]

**Appendix A. Equipment Descriptions**

The Uppertone is a cable-based muscle strengthening system that can be accessed by both manual or power wheelchair users. The Uppertone is a stand-alone unit that allows participants to complete a myriad of upper extremity strength training exercises including chest press, back row, triceps extension/rickshaw, and bicep curl from his/her personal wheelchair. The Equalizer is a collection of accessible cable-based strength training machines that includes separate free-standing machines and components to complete each type of exercise (i.e. chest press, rowing, rikshaw, bicep curl). The Vitaglide is a manual or power wheelchair accessible machine that incorporates upper extremity movements similar to cross country skiing or rowing. The Vitaglide is a versatile exercise machine that includes both strengthening and cardiovascular endurance training components. The Endorphin Arm Ergometer is a cardiovascular endurance machine that is height-adjustable allowing for participants to complete the arm crank motion from his/her personal manual or power wheelchair.
